# Guanosine Prevents Spatial Memory Impairment and Hippocampal Damage Following Amyloid-β_1–42_ Administration in Mice

**DOI:** 10.3390/metabo12121207

**Published:** 2022-12-01

**Authors:** Victor Coelho, Luisa Bandeira Binder, Naiani Ferreira Marques, Leandra Celso Constantino, Gianni Mancini, Carla Inês Tasca

**Affiliations:** 1 Programa de Pós-Graduação em Neurociências, Centro de Ciências Biológicas, Universidade Federal de Santa Catarina, Florianópolis 88040, Brazil; 2 Programa de Pós-Graduação em Bioquímica, Centro de Ciências Biológicas, Universidade Federal de Santa Catarina, Florianópolis 88040, Brazil; 3 Departamento de Bioquímica, Centro de Ciências Biológicas, Universidade Federal de Santa Catarina, Florianópolis 88040, Brazil

**Keywords:** Alzheimer’s disease, guanosine, Aβ_1–42_, glutamate, hippocampus

## Abstract

Alzheimer’s disease (AD) is a progressive neurodegenerative illness responsible for cognitive impairment and dementia. Accumulation of amyloid-beta (Aβ) peptides in neurons and synapses causes cell metabolism to unbalance, and the production of reactive oxygen species (ROS), leading to neuronal death and cognitive damage. Guanosine is an endogenous nucleoside recognized as a neuroprotective agent since it prevents glutamate-induced neurotoxicity by a mechanism not yet completely elucidated. In this study, we evaluated behavioral and biochemical effects in the hippocampus caused by the intracerebroventricular (i.c.v.) infusion of Aβ_1–42_ peptide (400 pmol/site) in mice, and the neuroprotective effect of guanosine (8 mg/kg, i.p.). An initial evaluation on the eighth day after Aβ_1–42_ infusion showed no changes in the tail suspension test, although ex vivo analyses in hippocampal slices showed increased ROS production. In the second protocol, on the tenth day following Aβ_1–42_ infusion, no effect was observed in the sucrose splash test, but a reduction in the recognition index in the object location test showed impaired spatial memory. Analysis of hippocampal slices showed no ROS production and mitochondrial membrane potential alteration, but a tendency to increase glutamate release and a significant lactate release, pointing to a metabolic alteration. Those effects were accompanied by decreased cell viability and increased membrane damage. Guanosine treatment prevented behavioral and biochemical alterations evoked by Aβ_1–42_, suggesting a potential role against behavioral and biochemical damage evoked by Aβ in the hippocampus.

## 1. Introduction

Alzheimer’s disease (AD) is a progressive and irreversible illness involving a severe disturbance in memory and cognition, which inevitably results in the need for intensive care [1,2]. Amyloid-beta (Aβ) is a proteolytic fragment formed from the cleavage of the amyloid precursor protein (APP), a transmembrane glycoprotein. The formation of 40 to 42 amino acids Aβ peptides in their oligomeric and/or fibrillar forms leads to their accumulation and these, in turn, promote neurotoxicity [3]. Synaptic and mitochondrial dysfunctions are observed both in transgenic AD models [4] and in models that mimic the early stages of AD, obtained through intracerebroventricular (i.c.v.) infusion of Aβ peptides [5]. Although acute Aβ infusion does not trigger all the pathological features of AD, i.c.v. Aβ_1–40_ infusion induces inflammatory responses, and decreases brain-derived neurotrophic factor (BDNF) levels. Additionally, it causes oxidative stress and changes in glutamatergic transmission in the hippocampus that may be associated with cognitive impairment in learning and memory, and in depressive-like behaviour in mice [6,7,8,9,10]. However, Aβ_1–42_ was identified as the most neurotoxic and prevalent in the central nervous system (CNS) in severe cases of AD and during disease progression [11]. Therefore, our aim was to evaluate the effect of a neuroprotective strategy on early events of Aβ_1–42_-induced hippocampal toxicity.

Guanosine (GUO) is an endogenous guanine nucleoside that acts as an intercellular signalling molecule affecting multiple neural processes [12,13,14]. Guanosine can be released to the extracellular milieu from astrocytes by nucleoside transporters [15], or it is produced in the extracellular space by guanine nucleotide metabolism via membrane-bound ectonucleotidases activity [16]. In ischemic or hypoxic insults, guanosine levels increase [17], and it has been suggested to act as a glutamatergic transmission modulator [14,18], thus reducing excitotoxicity. Guanosine has been shown to exert neuroprotective effects, such as preventing ischemic brain damage [19,20]; prevention of behavioral deficits and mitochondrial dysfunction in traumatic brain injuries [21,22]; prevention of memory deficits; and prevention of anhedonic-like behavior and loss of mitochondrial Ca^2+^ homeostasis induced by Aβ peptides [6,23]. Additionally, guanosine exerts an antidepressant-like effect in mice [13].

Of fundamental importance, guanosine shows several physiological effects, such as neurotrophic [24,25] and neurogenic [26], and favours antioxidant balance [22]. Guanosine was able to effectively decrease ROS levels, reduce mitochondrial swelling, and prevent mitochondrial membrane potential collapse in hippocampal slices subjected to oxygen-glucose deprivation [20].

Here we evaluated guanosine effects in an in vivo AD murine model assessing cell viability, oxidative, metabolic, and behavioural alterations during early events of Aβ_1–42_-induced hippocampal toxicity. The current study was the first to evaluate guanosine protective effects following 8 and 10 days of Aβ_1–42_ peptide i.c.v. infusion. Guanosine prevents alteration in the locomotor activity and the increased hippocampal ROS generation caused by Aβ_1–42_ (on the 8th day). Guanosine is also effective in preventing Aβ_1–42_-induced impairment in the hippocampal-dependent short-term spatial memory, increased damage to hippocampal slices, and metabolic alterations as increased lactate release (on the 10th day). Guanosine treatment prevented behavioural and biochemical alterations evoked by Aβ_1–42_.

## 2. Materials and Methods

### 2.1. Animals

Adult male Swiss mice (3 months old/40–50 g) provided by the animal facility from Universidade Federal de Santa Catarina (UFSC) were maintained in a 12-h cycle light/dark at 22 ± 1 °C (light phase started at 7:00 a.m.) in housing boxes (10 animals per box) with water and food ad libitum. Once received from the animal facility, mice were accommodated in the room for 10 days before experiments for acclimation. Animals were randomly selected from their housing boxes and allocated to treatment groups. The experimental procedures performed in this study followed “The ARRIVE Guidelines” published in 2010 and were approved by the Ethical Committee for Animal Research (CEUA/UFSC PP00955). Behavioral experiments started in the morning (8:00 a.m.). Animals were acclimatized with the handler before the beginning of the treatments to reduce stress. All experiments were designed to reduce the suffering and number of animals used. The study was not pre-registered, and no blinding was performed.

### 2.2. Drugs

Human Aβ_1–42_ (Tocris, Bristol, UK) was diluted in 0.1 M phosphate-buffered saline (PBS) in a stock solution (1 mg/mL) and then aggregated at 37 °C for 4 days and aliquots were stored at −20 °C. Guanosine (Sigma-Aldrich Brasil Ltda, Cat# G6752, Sao Paulo, Brazil) was freshly dissolved in saline (NaCl 0.9%) to 8 mg/kg (i.p.).

### 2.3. Amyloid-Beta Infusion

The aggregated form of amyloid-β_1–42_ (Aβ_1–42_) peptide (400 pmol/3 µL site) or PBS (3 µL/site) was administered intracerebroventricular (i.c.v.) according to [10]. Mice were briefly anesthetized with isoflurane 0.96% (0.75 CAM; Instituto BioChimico^®^, Rio de Janeiro, Brazil) using a vaporizing system (Isoflurane Vaporizer HB 4.3, HB Hospitalar Indústria e Comércio^®^, Sao Paulo, Brazil) and then gently retained by hand for the i.c.v administration. Under mild anaesthesia (only sufficient for loss of postural reflex), the needle was introduced unilaterally, not more than 2 mm into the brain, 1 mm to the right of the median point of each eye, and 1 mm posterior to a line drawn through the anterior base of the eye (used as an external reference). The exact location of the injection site was only confirmed at the time of dissection or euthanasia of the animals. No incorrect infusion was detected, and all mice results were included in the statistical analysis.

### 2.4. Experimental Design

**Figure 1:**
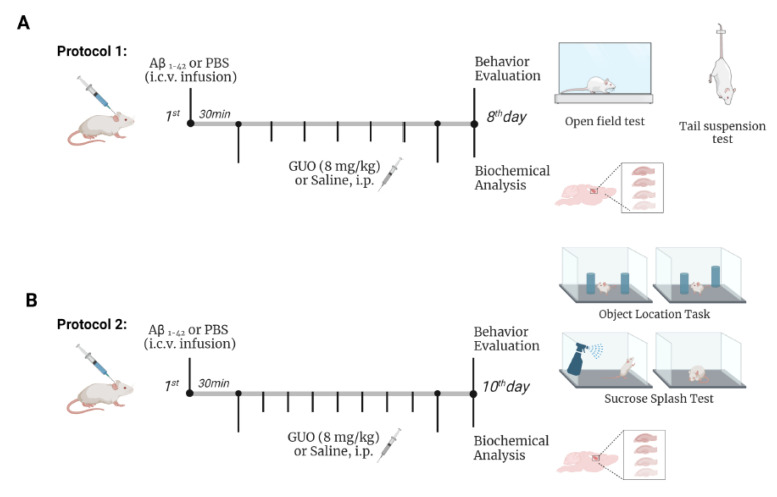
Overview of experimental protocols for behavioural and biochemical analysis in mice following Aβ_1–42_ i.c.v. infusion and guanosine treatment. Protocol 1: Mice were subjected to intracerebroventricular (i.c.v.) infusion of Aβ_1–42_ (400 pmol/3 uL site) or phosphate-buffered saline (PBS) infusion, and after 30 min received an intraperitoneal (i.p.) treatment of guanosine (GUO, 8 mg/kg) for 7 days. On the eighth day, mice were subjected to the open field test and to the tail suspension test, euthanized, and hippocampal slices assessed for ROS production and slice viability (MTT reduction and PI incorporation) (**A**). Protocol 2: On the tenth day after treatments, mice were subjected to the sucrose splash test and to the object location test. Animals were euthanized and hippocampal slices were assessed for ROS production, mitochondrial membrane potential, glutamate and lactate efflux, and slice viability (**B**).

To study the effects of guanosine on the molecular changes induced by Aβ_1–42_, animals were treated intraperitoneally (i.p.) with guanosine 8 mg/kg per day. On the first day, guanosine was administered 30 min after the i.c.v. infusion of Aβ_1–42_ or PBS, and treatment was carried out daily [10]. Control animals were treated with the vehicle saline (NaCl 0.9%; i.p.) for the same period. The treatment was carried out by the administration of 10 uL/g weight of the animal, both for guanosine and saline solutions. This study evaluated two-time courses of early Aβ_1–42_-induced neurotoxicity effects and guanosine modulation on behavioural and biochemical parameters: a first experimental group (Protocol 1) was analysed on day 8 following Aβ_1–42_ infusion and daily guanosine treatment (Figure 1A); and a second experimental group (Protocol 2) was analysed on day 10 following Aβ_1–42_ infusion and daily guanosine treatment (Figure 1B).

### 2.5. Behavioral Analysis

Mice subjected to Protocol 1 were tested for depressive-like behavior in the tail suspension test (TST) followed by the open field test (OFT) on day 8 following treatments (Figure 1A). In Protocol 2, independent animal cohorts were tested for anhedonic-like behavior or short-term spatial memory, assessed in the sucrose-splash test (SST), and in the object relocation test (ORT), respectively, at the day 10 following treatments (Figure 1B). Behavioral tests were carried out between 8:00 a.m. and noon, and they were scored by the same rater in an observation sound-attenuated room under low-intensity light (12 lux), where the mice had been habituated for at least 1 h before the beginning of the tests. Behavior was monitored through a video camera positioned above the apparatuses, and the videos were later analyzed with the ANYMaze^®^ (Stoelting Co., Wood Dale, IL, USA) video tracking system. The apparatus was cleaned with 10 % ethanol between animals to avoid odor clues.

### 2.6. Tail Suspension Test (TST)

Animals were subjected to TST, according to [27]. Mice both acoustically and visually isolated were suspended 50 cm above the floor by their tails with adhesive tape and placed approximately 1 cm from the tip of the tail. The total duration of mice immobility time induced by tail suspension was measured for 6 min [28].

### 2.7. Open-Field Test (OFT)

To assess the locomotor activity in order to ensure an antidepressant-like effect instead of an alteration of locomotor activity, mice were subjected to OFT after the TST. The apparatus consisted of a wooden box measuring 40 × 60 × 50 cm. The floor of the arena was divided into 12 equal squares. The number of squares crossed with all paws (crossings) was recorded for 6 min [29].

### 2.8. Sucrose Splash Test (SST)

On the 10th day following treatment, animals were subjected to the sucrose splash test, carried out as previously described [6]. SST consisted of sprayings a 10% sucrose solution on the dorsal coat of a mouse placed individually in clear Plexiglas boxes (9 × 7 × 11 cm). After applying sucrose solution, the latency and time spent grooming were recorded for a period of 5 min. The apparatuses were cleaned with a solution of 10 % ethanol between tests to hide the animal’s clues.

### 2.9. Object Location Task (OLT)

The short-term spatial memory of mice was assessed using the object location task as described [6]. The task consisted of two 5 min sessions (training and test) separated by a 90 min interval. In the training session, mice were placed in the center of the open field with two identical objects for 5 min, and object exploration was recorded using a stopwatch when mice sniffed, whisked, or looked at the objects from no more than 1 cm away. After 90 min, one object was moved to a new location, and the time spent by the animals exploring the objects in new (moved) and initial (familiar) locations was recorded over 5 min (test session). Objects were thoroughly cleaned with 10 % ethanol after each trial to minimize the presence of olfactory trails. To analyze the cognitive performance, a discrimination index of location was calculated as (T moved × 100)/(T moved + T familiar), where T moved is the time spent exploring the displaced object and T familiar is the time spent exploring the non-displaced object.

### 2.10. Biochemistry Analysis

#### 2.10.1. Preparation of Brain Slices

On the 8th (protocol 1, Figure 1A) or 10th day (protocol 2, Figure 1B) following treatments, animals were euthanized by decapitation and the whole brain was quickly removed and placed on an ice-cold wetted plate. Hippocampi were rapidly dissected in ice-cold Krebs-Ringer Buffer (KRB, composition in millimolar: NaCl 122, KCl 3, MgSO_4_1.2, CaCl_2_1.3, KH_2_PO_4_ 0.4, NaHCO_3_ 25, and D-glucose 10, bubbled with 95% CO_2_/5% O_2_ up to pH 7.4). Hippocampi were sliced (0.4 mm) using a McIlwain Tissue Chopper (The Mickle Laboratory Engineering Co., Ltd., England-RRID: SCR_015798, Guildford, Surrey, UK) and separated in KRB at 4 °C. After sectioning, three slices per well were incubated in 1 mL of KRB for 30 min, at 35 °C, for metabolic recovery before starting the experiment.

#### 2.10.2. Cellular Viability Evaluation

Cellular viability of the hippocampal slices was determined through the reduction assay of 3-(4,5-dimethylthiazol-2-yl)-2,5-diphenyltetrazolium bromide (MTT-Sigma). Slices were quantified by measuring the reduction of MTT to dark violet formazan, a product of mitochondrial dehydrogenases. Slices were incubated with MTT (0.5 mg/mL) in a KRB buffer for 20 min at 35 °C. The medium was removed, and the precipitated formazan was solubilized with 0.2 mL of DMSO for 30 min. After the removal of slices, the resulting coloured compound was quantified by a spectrophotometer TECAN ^®^ (Tecan Group Ltd., Mannedorf, Switzerland), equipment from the Laboratório Multiusuário de Estudos em Biologia at the Universidade Federal de Santa Catarina (LAMEB/UFSC) at a wavelength of 550 nm. Absorbance was used as an index of cellular viability [30].

#### 2.10.3. Propidium Iodide Incorporation

Cellular membrane integrity was assessed by evaluating the incorporation of the fluorescent dye propidium iodide (PI). PI is a polar compound that enters only cells with damaged membranes. Once inside the cells, PI complexes with DNA and emits an intense fluorescence. After the recovery period, slices were incubated with 7 μg/mL of PI for 30 min at 35 °C and then washed with KRB [31]. Fluorescence was measured in a fluorescence microplate reader (TECAN^®^, Mannedorf, Switzerland) using 495 and 630 nm as wavelengths of excitation and emission, respectively. Results were obtained as relative fluorescence units (RFU) from individual experiments, and the RFU values were normalized by percentage relative to the control group.

#### 2.10.4. Reactive Oxygen Species (ROS) Generation

The molecular probe 2,7-dichlorofluorescein diacetate (DCFH-DA, Sigma- Aldrich, St Louis, MO, USA) was used to measure ROS. DCFH-DA is a cell-permeable non-fluorescent probe, which is de-esterified intracellularly to the nonfluorescent form 2′,7′-dichlorofluorescein (DCFH). DCFH reacts with intracellular ROS and turns to 2′,7′-dichlorofluorescein (DCF), a highly fluorescent green die. After the recovery period, hippocampal slices were loaded with 80 μM of DCFH-DA for 30 min at 35 °C [32]. Slices were then washed with KRB and fluorescence was measured in a fluorescence microplate reader TECAN^®^ (LAMEB/UFSC) using excitation and emission wavelengths of 480 and 525 nm, respectively. Results were obtained as relative fluorescence units (RFU) from individual experiments, and RFU values were normalized by percentage relative to the control group.

#### 2.10.5. Mitochondrial Membrane Potential (ΔΨm) Measurement

Mitochondrial membrane potential was measured using the fluorescent probe tetramethylrhodamine ethyl ester (TMRE, Sigma-Aldrich, St Louis, MO, USA). The extinction protocol was used so that the selected concentration of TMRE, mitochondria selective fluorescent dye, was enough to form aggregates. Under these conditions, once diffused into the mitochondria, a subsequent mitochondrial depolarization results in the release of the dye, increasing the fluorescence signal. Hippocampal slices were incubated with TMRE (100 nM) for 30 min at 35 °C [32]. Fluorescence was measured in a fluorescence microplate reader TECAN^®^ using wavelengths of excitation and emission of 550 and 590 nm, respectively. Results were obtained as relative fluorescence units (RFU) from individual experiments, and RFU values were normalized by percentage relative to the control group.

#### 2.10.6. L-[^3^H]Glutamate Release

After the recovery period (30 min), hippocampal slices were incubated in Hank’s balanced salt solution (HBSS), composition in millimolar: CaCl_2_ 1.3, NaCl 137, KCl 5.36, MgSO_4_ 0.65, Na_2_HPO_4_ 0.3, KH_2_PO_4_ 1.1, and HEPES 5. Glutamate uptake was assessed by adding 0.33 μCi/mL of L-[^3^H] glutamate (American Radiolabeled Chemicals^®^) and 100 μM unlabelled glutamate in a final volume of 300 μL for loading the intracellular pool of L-[^3^H]glutamate, as previously described by [33]. Glutamate uptake was stopped after 7 min at 35 °C through two washes with 1 mL of ice-cold HBSS. To induce glutamate release, slices were incubated in 300 µL of HBSS for 15 min, and the supernatant was collected to assess the amount of L-[^3^H] glutamate release. Previous studies from our laboratory showed similar results by using D-[^3^H] aspartate or L-[^3^H] glutamate release [31]. Slices were homogenized by incubation with 0.1% NaOH and 0.01% SDS, and lysate aliquots were used to determine the intracellular amount of L-[^3^H] glutamate. The intracellular and extracellular L-[^3^H] glutamate content was analysed by the Liquid Scintillation Analyzer PerkinElmer^®^, and the amount of L-[^3^H] glutamate release was expressed as a percentage of total L-[^3^H]glutamate.

#### 2.10.7. Protein Measurement

Protein content was evaluated by the method of Lowry et al. [34]. Bovine serum albumin 1 mg/mL (Sigma) was used as a standard.

### 2.11. Statistical Analysis

The normal distribution of the data was tested with the Shapiro-Wilks test, and when the distribution of the variables was normal, further analyses were carried out. Comparisons among treated and control groups were performed by two-way ANOVA, when appropriate, followed by Tukey’s post hoc test. Grubb’s test was used to detect outliers. The novel object recognition task was analysed by one-sample *t*-tests to determine whether the recognition index was different from 50% (random investigation). A value of *p* ≤ 0.05 was considered significant. Statistical analysis and graphics were designed using GraphPad Prism^®^ 8.0.1 software package (San Diego, CA, USA). Figures were created with BioRender.com and Servier Medical Art.

## 3. Results

### 3.1. Guanosine Prevents Aβ_1–42_-Induced ROS Production

**Figure 2:**
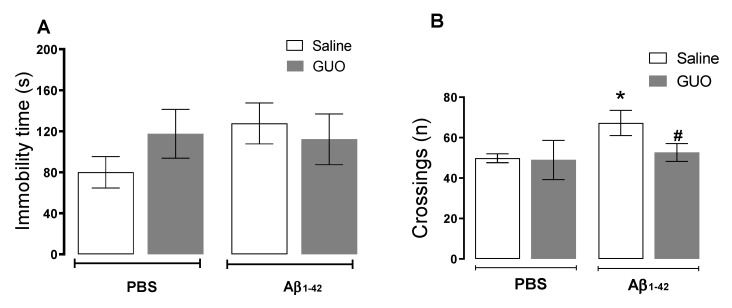
Evaluation of depressive-like behaviour in mice following guanosine treatment and Aβ_1–42_ i.c.v. infusion. Mice subjected to Aβ_1–42_ infusion (400 pmol/site, i.c.v.) received guanosine (GUO, 8 mg/kg, i.p.) for seven days. On the eighth day, mice were subjected to the tail suspension test (**A**) and to the open field test (**B**). Values expressed as mean + S.E.M (n = 6) * *p* ≤ 0.05 compared with the vehicle-treated group; # *p* ≤ 0.05 compared with Aβ_1–42_ -treated group. (Two-way ANOVA followed by Tukey’s test).

Regarding the results obtained in Protocol 1 (Figure 1A), we initially evaluated the effects of i.c.v. infusion of Aβ_1–42_ and guanosine treatment on the 8th day. Mice were tested in the tail suspension test (TST) followed by the open field test (OFT) (Figure 2A,B). Two-way ANOVA revealed no alterations in the immobility time in TST (*p ≥* 0.05). However, Aβ_1–42_ infusion produced an increased number of crossings in the OFT [(*F*_(1,17)_ = 7.22, (*p* = 0.0156)], and guanosine treatment was able to prevent this increased locomotor activity induced by Aβ_1–42_ [(*F*_(1,17)_ = 4.43, (*p* = 0.0468)].

**Figure 3:**
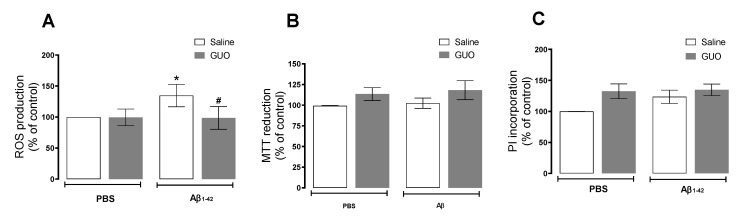
Guanosine prevents Aβ_1–42_-induced ROS production in mice hippocampal slices. Mice subjected to i.c.v. Aβ_1–42_ infusion (400 pmol/site) received guanosine (GUO, 8 mg/kg, i.p.) for seven days. On the eighth day, animals were euthanized, and hippocampal slices were analysed for ROS production (**A**), cellular viability through MTT assay (**B**), and cellular membrane damage through Propidium iodide (PI) incorporation (**C**). Values expressed as mean + S.E.M (n = 6–9) * *p* ≤ 0.05 compared with vehicle-treated group. # *p* ˂ 0.05 compared with Aβ_1–42_ group. (Two-way ANOVA followed by Tukey’s test).

Hippocampal slices of mice subject to Aβ_1–42_ infusion and guanosine (Protocol 1—Figure 1A) treatment were evaluated for ROS production and cell viability. ROS production analysis showed that vehicle-treated and guanosine-treated groups did not present any alteration in ROS production. Aβ_1–42_ infusion induced a 35% increase in ROS production when compared with the vehicle-treated group [(*F*_(1,15)_ = 5.052, (*p* = 0.0401)], and guanosine treatment prevented this effect [(*F*_(1,15)_ = 6.165, (*p* = 0.0253)] (Figure 3A). Regarding hippocampal viability, both guanosine treatment and Aβ_1–42_ infusion did not alter hippocampal slice viability (*p ≥* 0.05) (Figure 3B), or cellular membrane integrity (*p ≥* 0.05) (Figure 3C) at this time point.

### 3.2. Guanosine Prevents Aβ_1–42_-Induced Short-Term Spatial Memory Impairment

**Figure 4:**
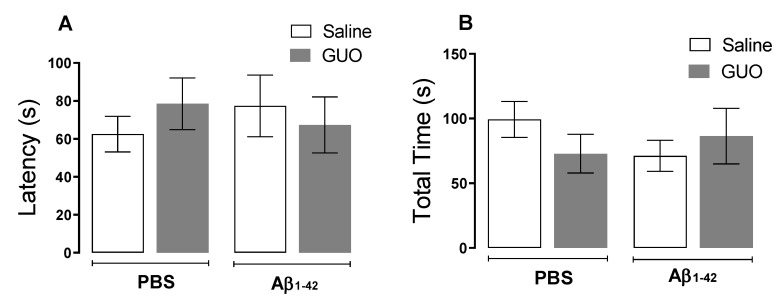
Evaluation of anhedonic-like behaviour in mice following guanosine treatment and Aβ_1–42_ i.c.v. infusion. Mice subjected to Aβ_1–42_ infusion (400 pmol/site) received guanosine (GUO, 8 mg/kg, i.p.) for nine days. On the tenth day, mice were subjected to the sucrose splash test and the latency to initiating grooming (**A**) and the total time spent in grooming activity (**B**) were measured. Values expressed as mean + S.E.M (n = 7–8). (Two-way ANOVA followed by Tukey’s test).

As previously demonstrated by our group, Aβ_1–40_ promoted an anhedonic-like behaviour and cognitive impairment [6]. Here our aim was to test Aβ_1–42_ effects on these behavioural paradigms on the 10th day, as the previous protocol showed no alteration in a depressive-like behaviour assessment. Anhedonic-like behaviour was tested through the sucrose splash test (SST) on the 10th day following treatments, as shown in the experimental design of Protocol 2 (Figure 1B). Statistical analysis indicated that Aβ_1–42_ infusion and guanosine treatment (8 mg/kg—9 days) did not significantly alter both latencies to initiating the grooming behaviour (*p ≥* 0.05) and the total time of grooming (*p ≥* 0.05) (Figure 4A,B).

**Figure 5:**
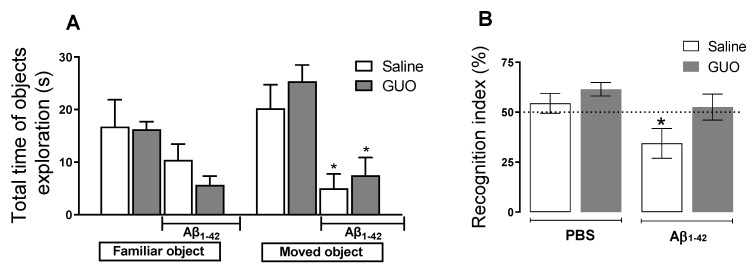
Guanosine prevents Aβ_1–42_-induced short-term spatial memory impairment in mice. Mice subjected to Aβ_1–42_ infusion (400 pmol/site) received guanosine (GUO, 8 mg/kg, i.p.) for nine days. On the tenth day, mice were subjected to the object location test and the total time of familiar and moved objects exploration were assessed (**A**) * *p* ≤ 0.05 compared with the vehicle- and GUO-treated group. The recognition index of location was calculated as the percentage of time exploring the moved object in relation to the time exploring the familiar object and * *p* < 0.05 compared to the hypothetical 50 % (dashed line) of random exploration (**B**). Values expressed as mean + S.E.M (n = 8–10). (Two-way ANOVA followed by Tukey’s test).

Additionally, mouse spatial memory was analysed in the object location test (OLT). Aβ_1–42_-treated mice reduced the total time of exploration of the object in a novel location (object 2) (Figure 5A). Aβ_1–42_ mice displayed impaired short-term spatial memory in the OLT, observed as a decreased index of recognition of the altered object (Figure 5B). Two-way ANOVA revealed a significant difference between the control group and the Aβ_1–42_ infused group [(*F*_(1,32)_ = 6.508, (*p* = 0.0157)]. Guanosine treatment prevented the decreased discrimination index induced by Aβ_1–42_ [(*F*_(1,32)_ = 4.916, (*p* = 0.0338)], suggesting the prevention of memory impairment in this hippocampal-dependent task.

### 3.3. Guanosine Prevents Aβ_1–42_-Induced Hippocampal Slice Damage

After behavioural analyses, an ex vivo analysis of hippocampal slices was performed by evaluating ROS production, mitochondrial membrane potential, slices cellular viability, as well as metabolic parameters (lactate and glutamate release).

**Figure 6:**
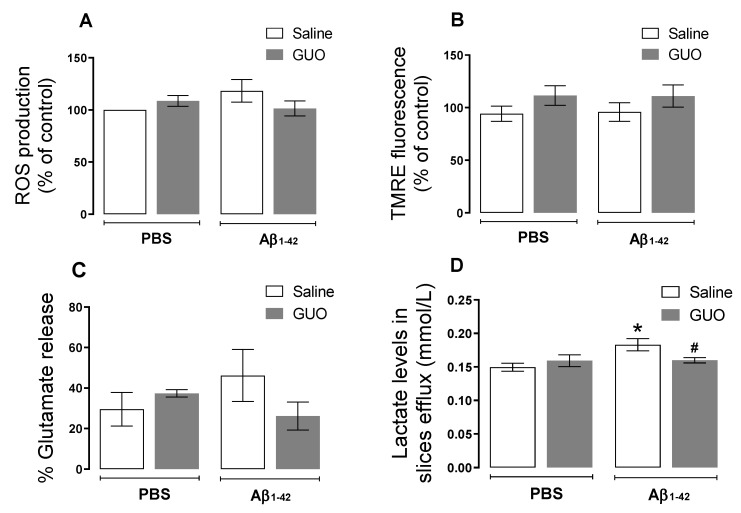
Biochemical evaluation of mice hippocampal slices following guanosine treatment and Aβ_1–42_ i.c.v. infusion. Mice subjected to Aβ_1–42_ infusion (400 pmol/site) received guanosine (GUO, 8 mg/kg, i.p.) for nine days. On the tenth day, animals were euthanized, and hippocampal slices were analyzed for ROS production (**A**), mitochondrial membrane potential through TMRE fluorescence assay (**B**), L-[^3^H] glutamate radioactivity in slices efflux was analysed to assess glutamate release (**C**), and lactate levels in slices efflux (**D**). Values expressed as mean + S.E.M (n = 6–8) * *p* ≤ 0.05 compared with vehicle-treated group. # *p* ˂ 0.05 compared with Aβ_1–42_ group. (Two-way ANOVA followed by Tukey’s test).

**Figure 7:**
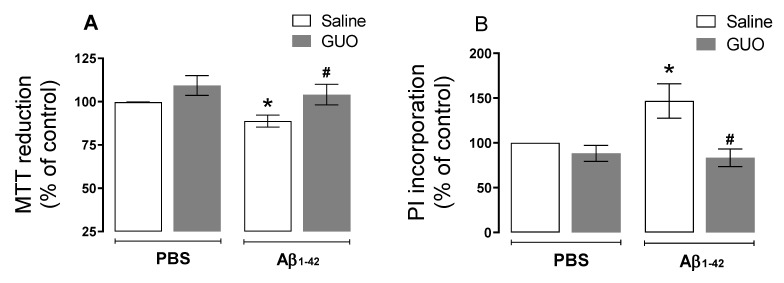
Guanosine prevents Aβ_1–42_-induced mice hippocampal slice loos of cell viability and membrane permeabilization. Mice subjected to Aβ_1–42_ infusion (400 pmol/site) received guanosine (GUO, 8 mg/kg, i.p.) for nine days. On the tenth day, animals were euthanized, and hippocampal slices were analysed for cellular viability through MTT assay (**A**), and membrane damage through PI incorporation (**B**). Values expressed as mean + S.E.M (n = 8–9) * *p* ≤ 0.05 compared with vehicle-treated group. # *p* ˂ 0.05 compared with Aβ_1–42_ group. (Two-way ANOVA followed by Tukey’s test).

Differently from the evaluation performed on the 8th day, here we observed no alteration in ROS production (*p ≥* 0.05) (Figure 6A). Additionally, no alteration in mitochondria membrane potential was observed (*p ≥* 0.05) (Figure 6B), and although not significant, glutamate release to the extracellular medium was slightly increased by Aβ_1–42_ (*p* = 0.1774)] (Figure 6C). Despite the fact that the oxidative status was not altered on day 10, here we observed increased lactate levels in the superfused slices medium (as an index of glycolytic activity) [(*F*_(1,18)_ = 4.982, (*p* = 0.0386)] (Figure 6D). Guanosine treatment was able to prevent this increase in lactate efflux. Slice viability analysis by MTT reduction and PI incorporation, showed decreased cell viability [(*F*_(1,27)_ = 3.337, (*p* = 0.0788)] and increased membrane permeabilization [(*F*_(1,29)_ = 4.280, (*p* = 0.0476)] evoked by Aβ_1–42_ (Figure 7A,B), and both cell viability and [(*F*_(1,27)_ = 7.414, (*p* = 0.0112)] membrane permeabilization [(*F*_(1,29)_ = 13.79, (*p* = 0.0009)] were prevented by guanosine treatment, suggesting the initial ROS production was the trigger to slices alterations thereafter.

## 4. Discussion

In the present study, i.c.v. infusion of Aβ_1–42_ promoted impairment in short-term spatial memory in mice subjected to the object location test, a hippocampal-dependent task. Additionally, an ex vivo evaluation of hippocampal slice functionality displayed a different profile regarding slice viability, ROS production, and metabolic alterations assessed in two-time points following Aβ_1–42_ infusion. The therapeutical strategy used, the intraperitoneal administration of the neuroprotective nucleoside guanosine, prevented spatial memory disruption, hippocampal ROS production, metabolic alteration, impairment in slice viability, and cell membrane damage.

The formation and aggregation of Aβ peptide oligomers are identified as the main cause of glutamatergic excitotoxicity in AD [35]. This event involves metabolic changes, inducing mitochondrial dysfunction, a process that is strongly related to the increased production of ROS, which can lead to cell death [36,37]. Whereas the administration of Aβ in the brains of rodents does not induce all the pathological aspects of AD, the intracerebroventricular (i.c.v.) infusion protocol is able to mimic the initial events of the disease, being a good model for biochemical and behavioral assessment of AD-related alterations. In the present study, we performed i.c.v. infusion of the Aβ_1–42_ peptide, identified as the most abundant and neurotoxic isoform in the CNS [11].

Several studies consider oxidative stress as a crucial event in the development of AD, with increased excessive ROS production promoting Aβ deposition, tau hyperphosphorylation, and subsequently synaptic and neuronal loss [38]. Our group and other researchers previously showed an acute oxidative effect on the mouse hippocampus 24 h after Aβ_1–40_ peptide i.c.v. infusion (400 pmol/site), where a significant increase in the production of ROS was observed [9,39]. Here, with i.c.v. infusion of Aβ_1–42_ peptide (at the same dose used for Aβ_1–40_), we also observed an increased ROS production even after seven days of Aβ_1–42_ administration (Figure 3A), and such effect was no longer observed in Protocol 2, at the 10th day of treatment (Figure 6A). Meanwhile, slice viability and cell membrane integrity were not initially altered (Figure 3B,C), but they were compromised when assessed later after an Aβ_1–42_ infusion (Figure 7). As previously mentioned, ROS production may be the trigger to decrease hippocampal slice viability observed thereafter.

We previously addressed the effects of Aβ_1–40_ infusion in mice, showing the induction of inflammatory responses, oxidative stress, and glutamatergic transmission changes. These alterations might be associated with cognitive impairment in learning and memory (evaluated 16 and 21 days after Aβ_1–40_) [6,10]. Additionally, Aβ_1–40_ promoted a reduction in hippocampal BDNF levels, and depressive-like behavior in mice (assessed ten days after Aβ_1–40_) [7,8]. In the present study, where we used the Aβ_1–42_ infusion, for an initial behavioral assessment, mice were subjected to TST to evaluate a depressive-like behavior and to the SST, to evaluate an anhedonic-like behaviour (Figure 2 and Figure 4). Unlike what was observed with Aβ_1–40,_ the Aβ_1–42_ peptide did not significantly alter the behavior of mice in both TST and SST.

Surprisingly, Aβ_1–42_ promoted an increased locomotor activity in the open field test (Figure 2B), which could resemble an anxiety-like behaviour, as shown by [40], which deserves further investigation. However, when evaluating the short-term spatial memory of mice, Aβ_1–42_ evoked a reduction in the time of exploration of the object in a new location (recognition index) in the OLT (Figure 5) suggesting a memory impairment induced by Aβ_1–42_. Such an effect was also previously observed with the protocol of Aβ_1–40_ infusion, though it was observed after 21 days of Aβ_1–40_ administration [6]. These observations point to the idea that although these different metabolic products of APP processing may have selective effects, memory impairment seems to be a similar outcome of i.c.v. administration of Aβ peptides.

A previous study from Souza da Silva et al., [23] also reported impairment in short-term memory by assessing the recognition of a novel object after 24 h, 7, and 14 days following Aβ_1–42_ oligomers i.c.v. infusion. However, metabolic alterations were assessed only 48 h after Aβ_1–42_ oligomers infusion and showed a partially compromised mitochondria activity. Here, on the 10th day following Aβ_1–42_ i.c.v. infusion, we analyzed hippocampal slices and observed no alteration in ROS production and mitochondrial membrane potential; however, there was a tendency in increasing glutamate release, and a significantly increased content of lactate (a glycolytic product) was found in the slice supernatant (Figure 6). Glutamate measurement in the extracellular space is usually interpreted as an excitotoxicity index, but considering it is also a substrate for oxidative metabolism, increasing tricarboxylic acid cycle activity, its accumulation in the extracellular space may also be related to a decreased oxidative metabolism [41,42,43,44]. However, glutamate fate and metabolism still need clarification in this Aβ_1–42_–induced toxicity protocol.

Additionally, considering the astrocytic-neuron lactate shuttle hypothesis [45], it is feasible that Aβ_1–42_-induced neurotoxicity involves a metabolic shift from oxidative to glycolytic profile. However, studies in humans have shown a decreased glucose metabolism associated with amnestic mild cognitive impairment and AD-related dementia [38,46]. Therefore, a more detailed metabolomic evaluation will be further necessary to accompany Aβ-induced metabolic shifts.

The nucleoside guanosine was the therapeutic strategy used in this study since it is a neuroprotective agent known to modulate glutamate excitotoxicity [12,14], by interacting with purinergic P1 adenosine A_1_/A_2A_ receptors heteromers [47], and/or calcium-activated potassium channels [48]. Guanosine was effective in preventing ROS production (Figure 3A) and increased lactate efflux (Figure 6D), and the loss of hippocampal viability (Figure 7) that accompanied memory impairment (Figure 5) in a hippocampal-dependent memory task [49,50]. Although guanosine is known to exert neuroprotection against glutamate toxicity-related disease models, such as seizures, ischemia, oxidative damage, and Parkinson’s disease [14], few studies have analyzed the effects of guanosine on AD models. In vitro, SH-5YSY neuroblastoma cells showed that guanosine displayed a protective effect against ROS production and apoptotic cell death induced by Aβ [51]. In vivo, guanosine treatment prevents the cognitive deficit and anhedonic-like behavior induced by Aβ_1–40_ in mice [6], and promotes presynaptic mitochondrial calcium homeostasis following Aβ_1–42_ oligomers i.c.v. infusion in mice [23]. The current study was the first to evaluate guanosine protective effects following 8 to 10 days of Aβ_1–42_ peptide i.c.v. infusion, the Aβ isoform most abundant in the brain and involved in triggering glutamatergic excitotoxicity and oxidative, metabolic, and behavioural distress. It is important to highlight that here guanosine did not promote any unsafe effects *per se*, and it was able to counteract the Aβ_1–42_-induced harmful behavioural and biochemical effects. Altogether, our study reinforces the neuroprotective effects of guanosine and offers some important insights into the time course of Aβ_1–42_-induced neurotoxicity in the hippocampus, following i.c.v. infusion.

## 5. Conclusions

**Figure 8:**
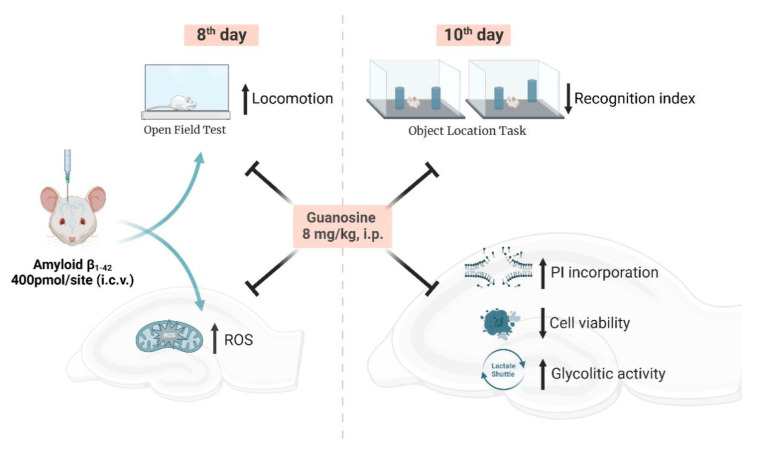
Schematic summary of neuroprotective effects of guanosine on behavioural and biochemical alterations in mice hippocampus following Aβ_1–42_ i.c.v. infusion. Guanosine prevents alteration in the locomotor activity and the initial hippocampal ROS generation caused by Aβ_1–42_ (eighth day). Guanosine is effective in preventing Aβ_1–42_-induced impairment in the hippocampal-dependent short-term spatial memory and damage to hippocampal slices (tenth day). The mechanism of action of neuroprotective effect of guanosine seems to involve modulation of glutamate and lactate transport, pointing to a metabolic modulation that prevents oxidative damage.

The present study demonstrated that guanosine prevents the initial production of ROS caused by Aβ_1–42_ (following eight days of Aβ_1–42_ peptide i.c.v. infusion), and is effective in preventing the damage to hippocampal slices at the same time it prevents Aβ_1–42_-induced impairment of short-term spatial memory (following ten days of Aβ_1–42_ peptide i.c.v. infusion). Additionally, guanosine modulates glutamate and lactate efflux from hippocampal slices (Figure 8). Overall, these findings provide support for the premise that guanosine has protective effects against memory impairment and brain damage and should be further investigated to better elucidate AD pathophysiology.

## Data Availability

All data generated or analysed during this study are included in this published article.
